# Retinal Artery and Vein Occlusions Successfully Treated with Hyperbaric Oxygen

**DOI:** 10.5811/cpcem.2019.7.43017

**Published:** 2019-09-25

**Authors:** Daniel R. Johnson, Jeffrey S. Cooper

**Affiliations:** University of Nebraska Medical Center, Department of Emergency Medicine, Omaha, Nebraska

## Abstract

We present six cases of central retinal artery occlusion (CRAO) and central retinal vein occlusion (CRVO) that we recently treated with hyperbaric oxygen (HBO_2_). Patients in three of the six cases, including the CRVO case, experienced near to complete restoration of their vision. Another case had marked improvement. Our findings are similar to other case studies with approximately 65–70% improvement in patients treated for CRAO. Physicians should be aware that rapid referral of CRAO and CRVO patients to HBO_2_ is efficacious. Such patients should be placed on 100% oxygen by non-rebreather mask as soon as the diagnosis is suspected, pending transportation to HBO_2_.

## INTRODUCTION

Central retinal artery occlusion (CRAO) is an emergent condition, typically presenting as sudden painless visual loss.^1^ Risk factors for CRAO include giant cell arteritis, atherosclerosis, atrial fibrillation, and thromboembolic disease. Permanent eye injury usually occurs after two hours of the occlusive event. CRAO is caused by embolism in the central retinal artery. Branch artery occlusion can also occur in the smaller (ciliary) branches of this artery. The occlusion leads to ischemia in the retina leading to pallor and the classic finding of “cherry red” macula due to increased visualization of the macula through the retina.^1^

Treatment methods include ocular massage to dislodge the embolus by creating a pressure differential, medications to decrease intraocular pressure (IOP), increasing partial pressure of carbon dioxide to cause retinal artery dilation with carbogen, a mixture of 95% oxygen and 5% carbon dioxide, intra-arterial fibrinolysis or systemic thrombolytics, and in extreme cases anterior chamber paracentesis to create an acute drop in IOP to dislodge the embolism. None of these interventions have demonstrated significant success.^2^ Recently hyperbaric oxygen (HBO_2_) has been approved by the Undersea and Hyperbaric Medical Society (UHMS) for treatment of CRAO due to evidence of significant efficacy.^3^

Central retinal vein occlusion (CRVO) has similar presenting symptoms and pathology to CRAO. CRVO is acute monocular vision loss from occlusions of the central retinal vein resulting in edema and ischemia to the retina. This occurs due to the central retinal artery and vein being the principal blood supply and drainage for the retina. Risk factors include hypertension (HTN), diabetes mellitus (DM), glaucoma and hypercoagulable conditions.^4^ Treatment considerations are similar to CRAO; however, anti-vascular endothelial growth factor medications are sometimes indicated to prevent macular edema and neovascularization, which can lead to glaucoma. HBO_2_ has been a proposed treatment modality for CRVO due to the similarities in pathology when compared to CRAO.

In this article we will discuss six cases, four of which were CRAO, one a branch retinal artery occlusion (BRAO), and one of CRVO. All were treated with HBO_2_ with improvement. The protocols used in these cases are from the 13^th^ edition of the UHMS HBO_2_ therapy guidelines. Treatments were generally 90 minutes at prescribed pressure, which was 2.5–2.8 atmospheres absolute with air breaks and additional time for compression and decompression.

## CASE SERIES

### Case One

Patient A, a 73-year-old female with a past medical history of coronary artery disease (CAD), congestive heart failure (CHF), atrial fibrillation, aortic and mitral valve replacement, HTN, hyperlipidemia (HPL), and DM, presented from an outside facility with acute painless monocular vision loss when bending over to pick something up. The patient was initially evaluated by an ophthalmologist and diagnosed with CRAO. Visual acuity (VA) in the right eye oculus dextrus (OD) was not testable due to blindness; left eye oculus sinister (OS) had baseline visual acuity of 20/25. The patient underwent HBO_2_ treatment within 13 hours of last known normal time and tolerated five treatments with improvement in the peripheral visual field; however, the central visual field defect remained.

### Case Two

Patient B, a 59-year-old male with a past medical history of HTN, DM, and aortic stenosis with mechanical valve replacement, was admitted for CRAO diagnosed at an outside facility. He underwent HBO_2_ treatment within 23 hours of initial injury. VA of OD was zero, OS 20/25. The patient underwent five HBO_2_ treatments per protocol and regained some peripheral vision during the hospitalization and upon discharge was able to count fingers (Table).

### Case Three

Patient C, a 39-year-old female with a past medical history of anxiety and hypothyroidism, presented with left sided retrobulbar headache. She was diagnosed with paraclinoid internal carotid artery aneurysm and underwent pipeline embolization with full symptomatic improvement and normal visual acuity upon discharge. She presented three days later and was found to have a left BRAO with initial visual acuity of 20/100 in the affected eye. She underwent HBO_2_ therapy within 10 hours of initial insult. After five HBO_2_ treatments her visual acuity returned to baseline (Table).

### Case Four

Patient D, a 73-year-old male, presented with a history of head injury at age 11 causing blindness in his left eye, peripheral vascular disease, HTN, chronic obstructive pulmonary disease, and DM. He had acute onset of visual loss in his right eye after waking from a nap. He was diagnosed with CRAO at an outside facility. His initial visual acuity could not be obtained due to blindness in both eyes. After three HBO_2_ treatments his visual acuity improved to 20/50 OD. The patient was unable to tolerate further HBO_2_ treatments due to confinement anxiety (Table).

### Case Five

Patient E, a 62-year-old female with a past medical history of HTN, carotid artery stenosis, CAD, and tobacco abuse, presented as a transfer from an outside facility due to right sided painless visual loss. HBO_2_ treatment was initiated 23 hours after initial symptom onset. The patient was only able to tolerate two and a half HBO_2_ treatments due to confinement anxiety. She was pretreated with lorazepam on her second HBO_2_ treatment unsuccessfully, and unfortunately declined further treatment. Visual acuity had improved from light perception to ability to visualize hand motion (Table).

**CPC-EM Capsule**

What do we already know about this clinical entity?

*Central retinal artery occlusion* (*CRAO) is an emergent condition. None of the generally suggested treatment interventions have demonstrated significant success until the recent use of hyperbaric oxygen (HBO_2_)*.

What makes this presentation of disease reportable?

*We report six cases, four CRAO, one branch retinal artery occlusion, and one central retinal vein occlusion with varied results and a discussion of the rationale for efficacy*.

What is the major learning point?

*HBO_2_ may salvage vision loss in retinal artery and vein occlusions*.

How might this improve emergency medicine practice?

*Rapid identification and referral to hyperbaric therapy is important to improve the chances of visual recovery in retinal artery and vein occlusions*.

### Case Six

Our final patient F, a 46-year-old male, presented with acute painless monocular visual loss in the left eye. Symptoms started 48 hours prior to presentation. This patient had a past medical history significant for HTN, DM, and HLD, and Sjogren’s syndrome with previous CRVO of the right eye with blindness. He was found to have a new CRVO in his left eye. He underwent 10 HBO_2_ treatments with near-complete improvement in visual acuity to 20/30 at discharge (Table).

## DISCUSSION

The above-mentioned cases had improvement in vision with HBO_2_ therapy for vaso-occlusive injury to the eye. Patients in three out of the six cases experienced near to complete restoration of their vision. Another case had marked improvement. Confinement anxiety was an issue in two cases. Our findings are similar to other case studies with approximately 65–70% improvement in patients treated for CRAO^3^ with an excellent result in CRVO.

While the eye is primarily supplied by the retinal artery, there is also some contribution by the choroidal vessels (ciliary arteries). Under normal circumstances, the choroidal supply is inadequate to support the retina; however, under hyperbaric conditions the choroidal circulation can supply the retina with adequate oxygen. This can allow the retina to survive until the retinal arterial (or venous) occlusion resolves via intrinsic thrombolytic mechanisms. HBO_2_ also ameliorates subsequent reperfusion effects and edema.^3^

## CONCLUSION

Hyperbaric oxygen has established a clear efficacy for treating CRAO. There have been multiple case reports with promising outcomes for CRVO as well.^4^–^6^ Both emergency physicians and ophthalmologists should be aware that rapid referral of CRAO and CRVO patients to HBO_2_ therapy is efficacious. Such patients should be placed on 100% oxygen by non-rebreather mask as soon as the diagnosis is suspected^7^ pending transportation to HBO_2_.

## Figures and Tables

**Table:** Summary of cases of sudden monocular blindness treated with hyperbaric oxygen.

Patient	Hours from vision loss to HBO_2_	Number of HBO_2_ treatments	Outcome	Comment
A	13	5	Some peripheral field improvement only	
B	23	5	Zero vision to finger counting	
C	10	5	Complete resolution	Branch retinal artery occlusion
D	9	3	Improved to 20/50	Confinement anxiety
E	23	2.5	From light perception to ability to visualize hand motion	Confinement anxiety
F	48	10	Near-complete resolution 20/30	Central retinal vein occlusion

*HBO_2_*, hyperbaric oxygen.
